# Intracerebral hemorrhage location and outcome among INTERACT2 participants

**DOI:** 10.1212/WNL.0000000000003771

**Published:** 2017-04-11

**Authors:** Candice Delcourt, Shoichiro Sato, Shihong Zhang, Else Charlotte Sandset, Danni Zheng, Xiaoying Chen, Maree L. Hackett, Hisatomi Arima, Jun Hata, Emma Heeley, Rustam Al-Shahi Salman, Thompson Robinson, Leo Davies, Pablo M. Lavados, Richard I. Lindley, Christian Stapf, John Chalmers, Craig S. Anderson, For the INTERACT2 Investigators

**Affiliations:** From The George Institute for Global Health and The University of Sydney (C.D., S.S., E.C.S., D.Z., X.C., M.L.H., E.H., R.I.L., J.C., C.S.A.); Royal Prince Alfred Hospital (C.D., L.D., J.C., C.S.A.), Camperdown, Australia; National Cerebral and Cardiovascular Center (S.S.), Osaka, Japan; Department of Neurology (S.Z.), West China Hospital, Sichuan University, Chengdu; Oslo University Hospital (C.S.), Norway; The University of Central Lancashire (M.L.H.), UK; Department of Preventive Medicine and Public Health (H.A.), Faculty of Medicine, Fukuoka University; Center for Cohort Studies (J.H.), Graduate School of Medical Sciences, Kyushu University, Fukuoka, Japan; Division of Clinical Neurosciences (R.A.-S.S.), Centre for Clinical Brain Sciences, University of Edinburgh; Department of Cardiovascular Sciences and NIHR Biomedical Research Unit for Cardiovascular Diseases (T.R.), University of Leicester, UK; Clínica Alemana de Santiago (P.M.L.), Facultad de Medicina Clinica Alemana Universidad del Desarrollo; Facultad de Medicina (P.M.L.), Universidad de Chile, Santiago; Westmead Hospital Clinical School (R.I.L.), Westmead, Australia; Centre de Recherche du Centre Hospitalier de l'Université de Montréal (CRCHUM) (C.S.), Département de Neurosciences, Université de Montréal, Canada; and The George Institute China (C.S.A.), Peking University Health Sciences Center, Beijing, China.

## Abstract

**Objective:**

To clarify associations between intracerebral hemorrhage (ICH) location and clinical outcomes among participants of the main phase Intensive Blood Pressure Reduction in Acute Cerebral Hemorrhage Trial (INTERACT2).

**Methods:**

Associations between ICH sites and poor outcomes (death [6] or major disability [3–5] of modified Rankin Scale) and European Quality of Life Scale (EQ-5D) utility scores at 90 days were assessed in logistic regression models.

**Results:**

Of 2,066 patients included in the analyses, associations were identified between ICH sites and poor outcomes: involvement of posterior limb of internal capsule increased risks of death or major disability (odds ratio [OR] 2.10) and disability (OR 1.81); thalamic involvement increased risks of death or major disability (OR 2.24) and death (OR 1.97). Involvement of the posterior limb of the internal capsule, thalamus, and infratentorial sites were each associated with poor EQ-5D utility score (≤0.7 [median]; OR 1.87, 2.14, and 2.81, respectively). Posterior limb of internal capsule involvement was strongly associated with low scores across all health-related quality of life domains. ICH encompassing the thalamus and posterior limb of internal capsule were associated with death or major disability, major disability, and poor EQ-5D utility score (OR 1.72, 2.26, and 1.71, respectively).

**Conclusion:**

Poor clinical outcomes are related to ICH affecting the posterior limb of internal capsule, thalamus, and infratentorial sites. The highest association with death or major disability and poor EQ-5D utility score was seen in ICH encompassing the thalamus and posterior limb of internal capsule.

**ClinicalTrials.gov registration:**

NCT00716079.

The prognosis for recovery from acute intracerebral hemorrhage (ICH) is strongly related to several radiologic criteria. These include hematoma volume and the degree of extension of blood into the ventricles and the subarachnoid space.^1–3^ Infratentorial location of ICH also predicts a higher likelihood of death or dependency.^1,4^ However, the relationship between specific locations of supratentorial ICH and outcome is poorly understood due in part to varying designs and definitions across studies.^5^ We examined associations of ICH location with clinical outcomes among participants of the main phase Intensive Blood Pressure Reduction in Acute Cerebral Hemorrhage Trial (INTERACT2).^6^

## METHODS

### Patients.

The INTERACT2 study was a randomized, open, multicenter, controlled trial with blinded outcome assessment conducted between 2008 and 2012.^6^ A total of 2,839 patients with imaging-confirmed ICH were randomly assigned to receive either early intensive blood pressure (BP)–lowering treatment (<140 mm Hg systolic BP goal) or the contemporaneous guideline-recommended BP management (<180 mm Hg systolic BP goal) within 6 hours of onset. Follow-up was to 90 days.

### Standard protocol approvals, registrations, and patient consents.

The study protocol was approved by the appropriate ethics committee at each participating site, and written informed consent was obtained from patients or appropriate surrogates. INTERACT2 is registered with ClinicalTrials.gov (NCT00716079).

### Measures.

Demographic and clinical characteristics were recorded upon patient enrollment. CT scans were performed according to standardized techniques (recommended slice thickness: 5–8 mm) at baseline and centrally analyzed for volume and location of ICH and the presence of intraventricular hemorrhage (IVH) extension.

Three neurologists reviewed all CT scans for detailed assessment of sites involved by each ICH. Defined sites were the caudate head, putamen/globus pallidus, thalamus, posterior limb of internal capsule, anterior limb of internal capsule, external capsule, any lobar region, and infratentorial. ICH could involve one or several sites. For small bleeds confined to any site, that site was classified as involved. For 3 well-delimited sites (caudate head, putamen/globus pallidus, and thalamus), involvement was defined as replacement of over one-third of the structure by blood (judgment made by assessor on CT scan axial slices). For the remaining sites, posterior limb of internal capsule, anterior limb of internal capsule, external capsule, any lobar location, and infratentorial any involvement led to these sites being classified as affected. Superficial supratentorial or lobar location was selected when structures other than deep structures (caudate, putamen/globus pallidus, thalamus, internal capsule) were involved. In large volume ICH, where the hemorrhage spanned the hemisphere from the ventricle to the cortex, location was rated as both deep, with affected sites listed, and lobar. ICH were then evaluated for their entire location, which could involve single or multiple sites. The term encompassing has been used to describe common patterns of ICH that involve one or more of the sites defined above.

Outcomes for these analyses were death or major disability as assessed by the modified Rankin Scale (mRS) (death, score of 6; major disability, score of 3–5) and health-related quality of life (HRQoL) as self-assessed by the patient or by a proxy responder using the European Quality of Life Scale (EQ-5D)^7^ questionnaire at 90 days. The EQ-5D uses a descriptive system defining the state of general health across 5 dimensions (mobility, self-care, usual activities, pain/discomfort, and anxiety/depression), with respondents rating their assessment of each as having no (score of 1), some or moderate (score of 2), or severe problems (score of 3). A utility score integrating ratings of the 5 dimensions into a single score was calculated by using population-based preference weights obtained from the UK population for each subscale. The utility score expresses HRQoL quantitatively as a fraction of perfect health: a score of 1 represents perfect health, 0 represents death, and negative scores represent health states considered worse than death; a score between 0.8 and 0.9 represents the average score in the general population.^8–10^ Both measures were used in the present analysis.

### Statistical analysis.

Baseline characteristics of included and excluded patients were summarized by means and SD for normally distributed variables, medians and interquartile ranges (IQR) for skewed continuous variables, and numbers (%) for categorical variables. Between-group differences were assessed using the χ^2^ test for categorical variables and the Wilcoxon test for continuous variables. Associations between ICH sites of involvement and death or major disability, poor HRQoL in each domain (grades of some to moderate or severe vs no problems in the corresponding dimensions), and poor overall HRQoL (utility score ≤0.7 [dichotomized by median]) were assessed in multivariable logistic regression models, adjusted for potential confounders. The adjusted variables included age, female sex, China region of recruitment, history of ischemic stroke and diabetes mellitus, medication history of antihypertensives, antithrombotics, and lipid-lowering agents, NIH Stroke Scale (NIHSS) score (≥11 vs <11 [dichotomized by median]), systolic BP, onset to CT time (log-transformed), baseline hematoma volume (log-transformed), IVH, allocation to intensive BP lowering, laterality (left vs right), and proxy response for the HRQoL models. Each individual site (caudate head, putamen/globus pallidus, thalamus, external capsule, anterior limb of internal capsule, posterior limb of internal capsule, lobar, and infratentorial) was included in the multivariable model to identify independent relationships between specific sites and outcome. The same analysis was repeated without adjusting for NIHSS. Also, common patterns of entire ICH encompassing one or multiple sites were included in a multivariable model adjusted for the same variables except individual sites. A standard level of significance (*p* < 0.05) was used and the data are reported with odds ratios (OR) and 95% confidence intervals (CI). All analyses were performed using SAS software version 9.3 (SAS Institute, Cary, NC).

## RESULTS

Among the 2,829 INTERACT2 participants, 2,066 (73%) were included in the mortality and functional outcome analyses with available baseline DICOM format CT and information on outcome (mRS) at 90 days (figure e-1 at Neurology.org). Table 1 shows the baseline characteristics of included and excluded patients; the former had smaller ICH (median 10.7 [IQR 5.6–18.7] mL vs 12.0 [6.3–23.9] mL) and were more likely to answer the EQ-5D questions themselves (fewer proxy responders, indicative of less severe ICH).

**Table 1:**
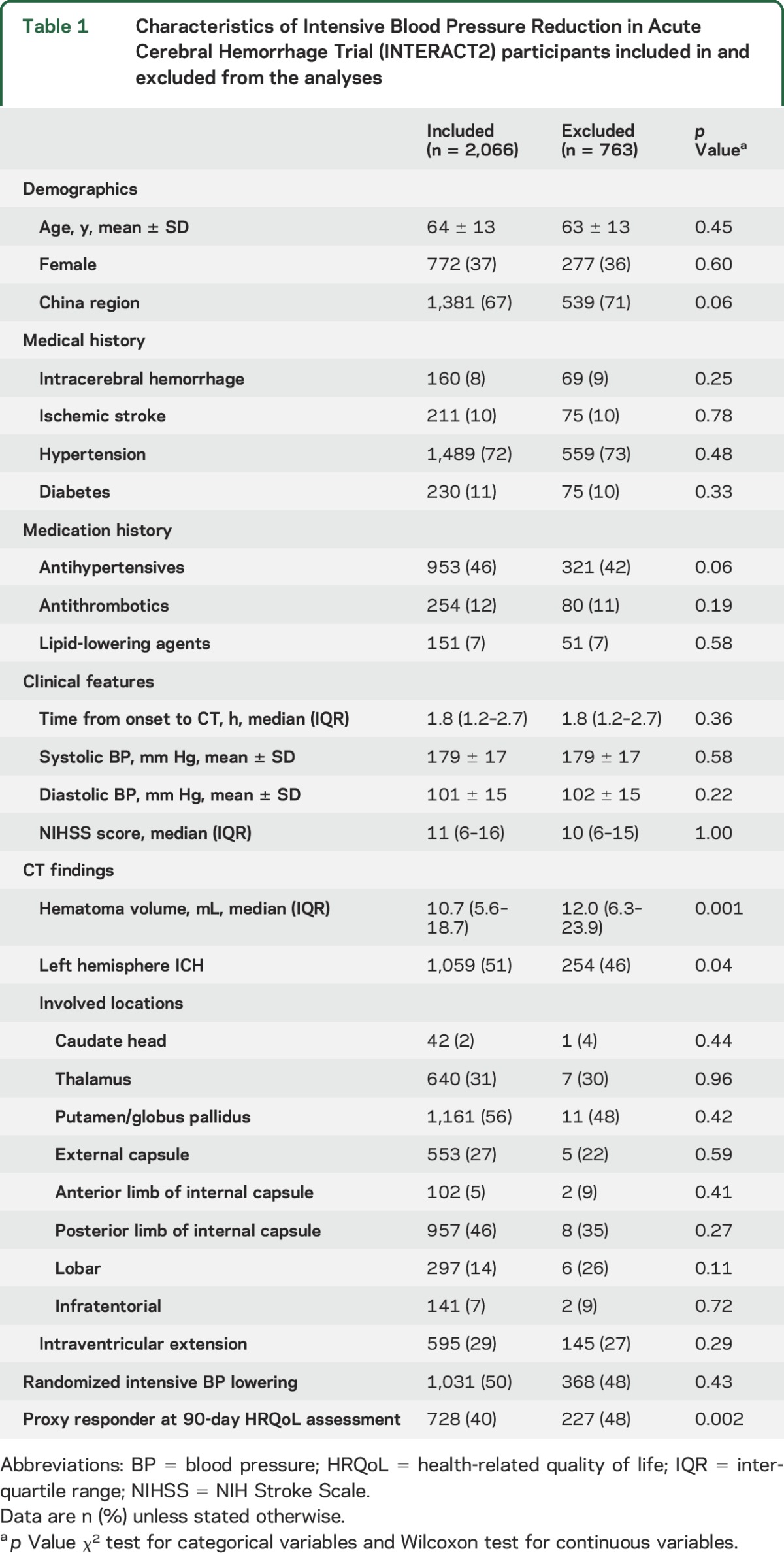
Characteristics of Intensive Blood Pressure Reduction in Acute Cerebral Hemorrhage Trial (INTERACT2) participants included in and excluded from the analyses

Table e-1 shows the most common patterns of ICH. Entire ICH included 56 combinations of possible sites. Of these possible combinations, 42 (75%) were present in fewer than 10 patients, 6 (11%) in between 10 and 50 patients, and 8 (14%) in more than 130 patients (total n = 1,747, 85% of the total population). The most common patterns were putamen/globus pallidus alone (n = 342), thalamus and posterior limb of internal capsule (n = 339), posterior limb of internal capsule, putamen/globus pallidus, and external capsule (n = 236), thalamus alone (n = 181), lobar alone (n = 181), putamen/globus pallidus and posterior limb of internal capsule (n = 177), putamen/globus pallidus and external capsule (n = 153), and infratentorial alone (n = 138).

Table 2 shows associations between ICH sites of involvement and death or major disability at 90 days. In models adjusted for baseline differences, thalamic, lobar, and infratentorial involvement were associated with death: OR 1.97 (95% CI 1.18–3.29), 1.95 (1.21–3.15), and 2.45 (1.09–5.50), respectively. ICH involving the posterior limb of internal capsule was associated with major disability: OR 1.81 (95% CI 1.45–2.26). ICH involving the thalamus, posterior limb of internal capsule, and infratentorial was associated with the composite endpoint of death and major disability: OR 2.24 (95% CI 1.40–3.57), 2.10 (1.65–2.68), and 3.04 (1.68–5.50), respectively. The same model with all the included variables is shown in table e-2.

**Table 2:**
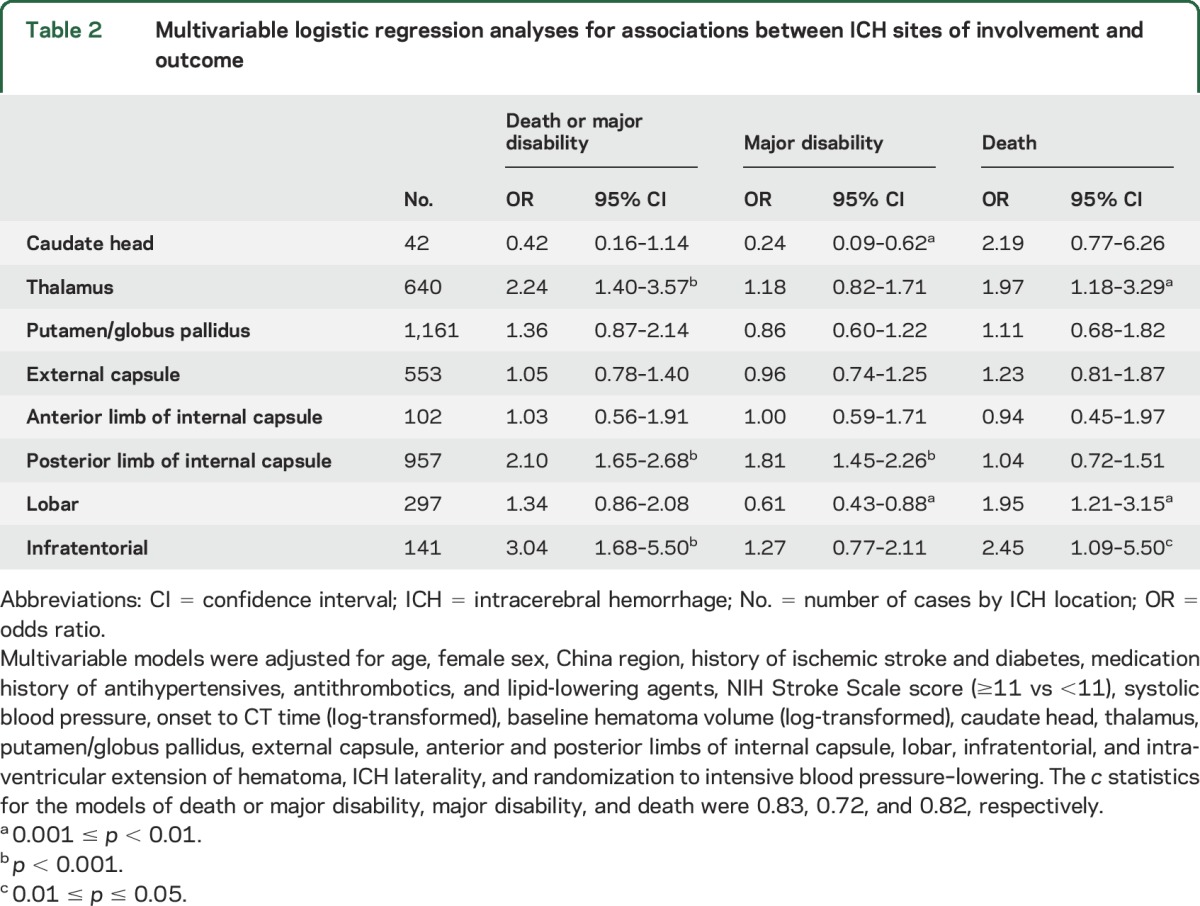
Multivariable logistic regression analyses for associations between ICH sites of involvement and outcome

For analysis of HRQoL, 251 patients who died within 90 days and 36 without EQ-5D information were excluded, leaving 1,779 patients for analysis of HRQoL (figure e-1). Table 3 shows associations between ICH location and HRQoL. After adjusting for potential confounders, thalamic OR 2.14 (95% CI 1.32–3.48), posterior limb of internal capsule OR 1.87 (95% CI 1.45–2.40), and infratentorial OR 2.81 (95% CI 1.52–5.20) sites were each associated with poor overall HRQoL (utility score ≤0.7). Posterior limb of internal capsule was associated with poor HRQoL across all domains. Thalamic and infratentorial sites were associated with poor HRQoL in the mobility, self-care, and usual activity domains.

**Table 3:**
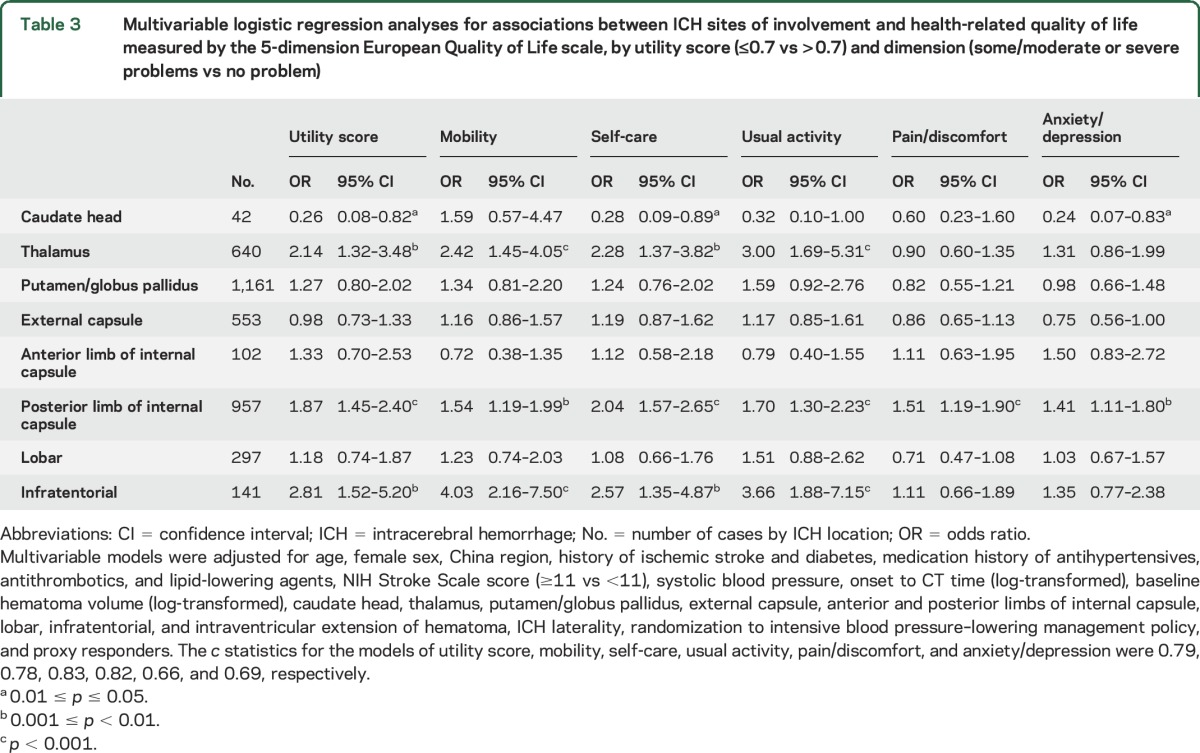
Multivariable logistic regression analyses for associations between ICH sites of involvement and health-related quality of life measured by the 5-dimension European Quality of Life scale, by utility score (≤0.7 vs >0.7) and dimension (some/moderate or severe problems vs no problem)

The same model with all the included variables is shown in table e-3. Tables e-2 and e-3 show that death, major disability, and poor HRQoL were also associated with older age, increased baseline neurologic severity (NIHSS), and baseline ICH volume. Similar results were found when the analyses were not adjusted for NIHSS (tables e-4 and e-5).

Table 4 shows associations between the most common patterns of entire ICH and death or disability at 90 days. After adjustment for potential confounders, ICH encompassing the thalamus and posterior limb of internal capsule were associated with death or major disability (OR 1.72 [95% CI 1.14–2.58]) and major disability (2.26 [1.58–3.23]). ICH encompassing the posterior limb of internal capsule and putamen/globus pallidus with or without additional involvement of the external capsule were associated with disability (OR 1.69 [95% CI 1.13–2.54] and 1.52 [1.04–2.21]).

**Table 4:**
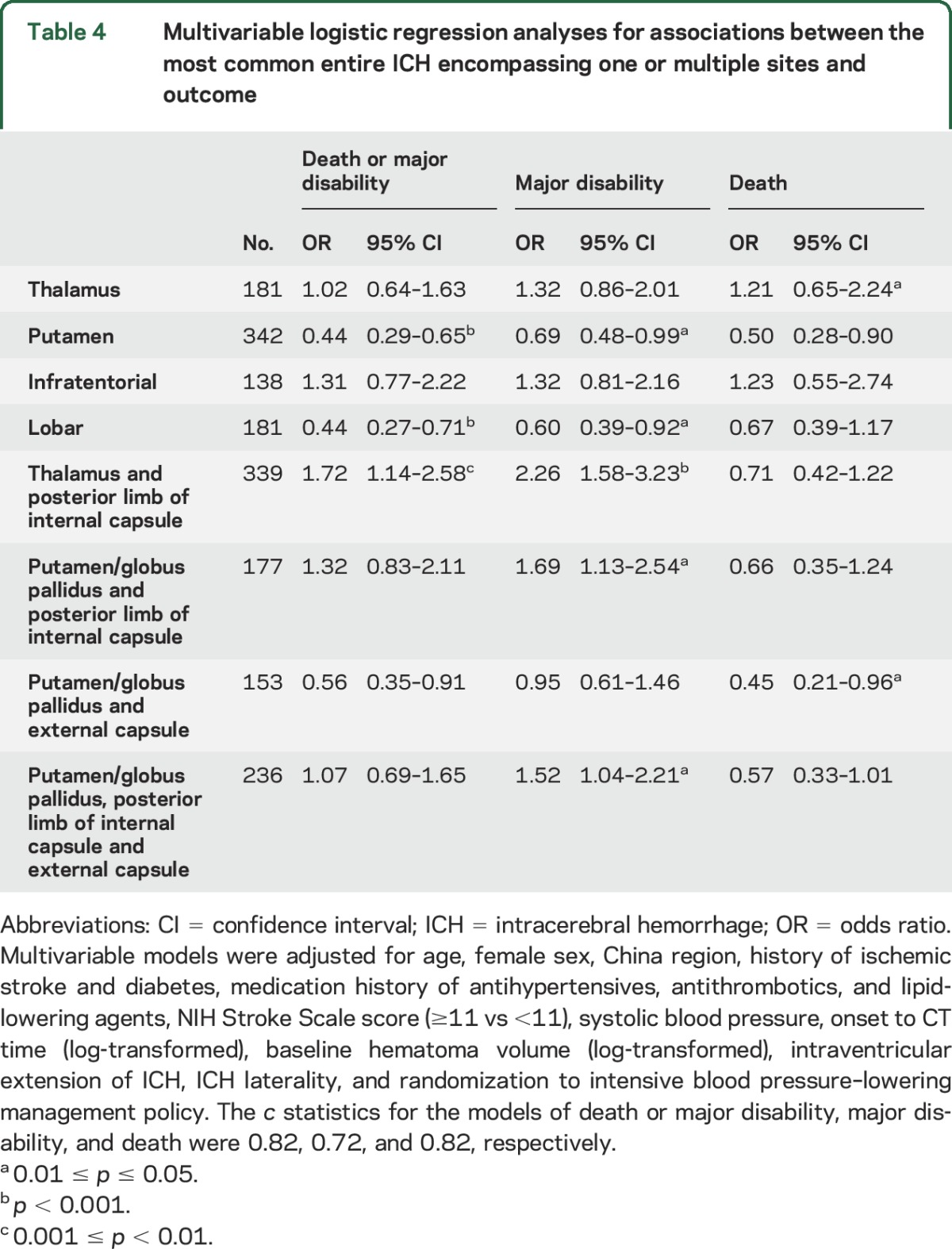
Multivariable logistic regression analyses for associations between the most common entire ICH encompassing one or multiple sites and outcome

Table 5 shows associations between the most common patterns of entire ICH and HRQoL. After adjustment for potential confounders, ICH encompassing the thalamus and posterior limb of internal capsule were associated with poor EQ-5D utility score (≤0.7 [median]; OR 1.71 [95% CI 1.12–2.60]) and poor HRQoL in the self-care, usual activity, and pain/discomfort domains: OR 2.08 (95% CI 1.34–3.22), 2.26 (1.42–3.60), and 1.78 (1.21–2.62), respectively. Thalamic ICH was associated with poor HRQoL in the usual activity and pain/discomfort domains: OR 1.75 (95% CI 1.04–2.95) and 1.58 (1.00–2.49; *p* = 0.049). Infratentorial ICH was associated with poor HRQoL in the mobility and usual activity domain: OR 1.78 (95% CI 1.02–3.10) and 1.86 (1.06–3.26). ICH encompassing the putamen/globus pallidus, posterior limb of internal capsule, and external capsule were associated with poor HRQoL in the pain/discomfort domain: OR 1.65 (95% CI 1.10–2.47).

**Table 5:**
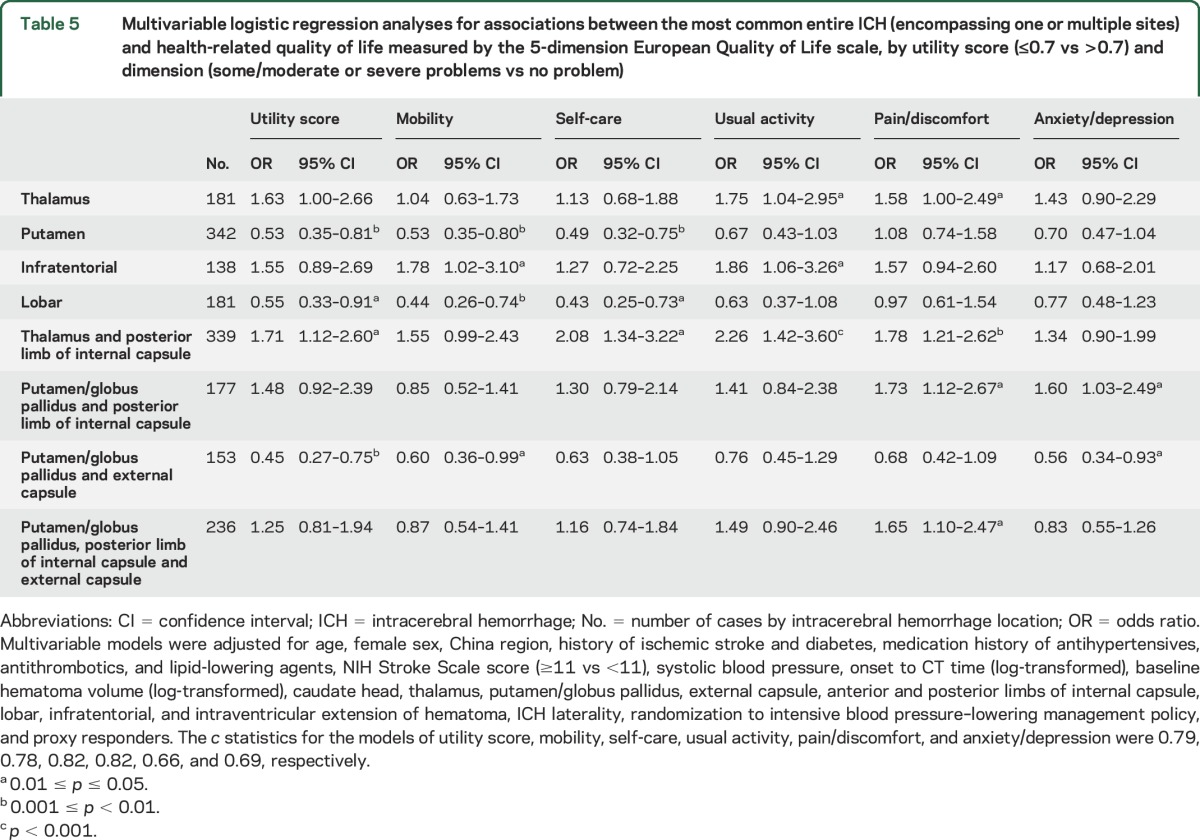
Multivariable logistic regression analyses for associations between the most common entire ICH (encompassing one or multiple sites) and health-related quality of life measured by the 5-dimension European Quality of Life scale, by utility score (≤0.7 vs >0.7) and dimension (some/moderate or severe problems vs no problem)

Table e-6 shows the hematoma volume by site of involvement and table e-7 shows the model fit statistics for tables 2 and 3.

## DISCUSSION

This secondary analysis of the INTERACT2 study dataset demonstrates strong associations between the location of ICH and clinically relevant, patient-centered outcomes. Any ICH that involves the posterior limb of internal capsule or thalamus increases the risks of death or disability and of disability alone. ICH involving the posterior limb of the internal capsule, thalamus, and infratentorial sites were each associated with poor HRQoL. In particular, involvement of the posterior limb of the internal capsule was strongly associated with poor outcomes across all HRQoL domains. Of the 8 common recognizable patterns of ICH, the striking characteristic is that all of these that involve the posterior limb of internal capsule are associated with major disability. Isolated thalamic hemorrhage and ICH encompassing the thalamus and posterior limb of internal capsule are associated with poor HRQoL. These data indicate that damage to the capsular pyramidal tracts is particularly disabling, while ICH involving the thalamus and posterior fossa are more likely to be fatal. In contrast, ICH confined to a lobar site seems to have a relatively benign prognosis when adjusted for hemorrhage volume. However, this does not necessarily mean that outcome after a capsular ICH is worse than outcome after a lobar ICH, as mean hemorrhage volume in lobar ICH is large by comparison with deep ICH and volume itself is an important predictor of outcome.^1^ When looking at ICH that involved a lobar site and one or more other sites, there is an increased risk of death. This is likely to reflect the fact that these are extensive ICH spanning from deep structures to the cortex. Finally, the analysis confirms well-recognized associations between older age,^11,12^ higher neurologic severity, ICH volume, and poor outcomes.^1^

There is increasing interest in the assessment of HRQoL after stroke, but there has been limited data on its association with ICH in specific brain locations. In the Factor VII for Acute Intracerebral Hemorrhage trial, deep compared to lobar location of ICH was associated with poor HRQoL,^13^ but no further assessment of anatomical structures was undertaken.

Damage to the posterior limb of the internal capsule on diffusion tensor tractography has been shown to be related to poor motor outcome and to disability in the ischemic stroke literature,^14^ while ICH in the thalamus has been shown to be associated with higher in-hospital mortality compared to ICH in other supratentorial locations.^15^ The pyramidal tracts pass through the posterior limb of the internal capsule location^16^ and pathology affecting motor function has predictable consequences for disability and HRQoL^17^ related directly to domains of mobility, usual activity, and self-care, and indirectly to the more subjective domains of pain/discomfort and anxiety/depression. Thalamic ICH is prone to leak blood into the ventricles^18^ and may extend to compress the brainstem with life-threatening consequences. Greater residual neurologic deficit may explain why survivors of ICH involving the posterior limb of internal capsule, thalamus, and infratentorium had worse overall HRQoL compared to those who recover from ICH in other locations. The finding that the domains of pain/discomfort and anxiety/depression showed no significant associations with thalamic involvement and a very weak association for the former with thalamic ICH in the multivariable models is surprising. Strokes in the thalamus have a high incidence of residual sensory disability and are traditionally associated with pain syndromes,^19^ while lesions of spinothalamic afferents to the posterior thalamus are associated with development of central pain.^20^ This effect seems small in ICH.

Strengths of our analysis include the assessment of HRQoL in a large group of participants with ICH with rigorous and structured central adjudication of images for determining ICH location. Limitations include selection bias from a clinical trial population where patients with severe ICH were purposefully excluded. From a practical point of view, the excluded patients were those with a very high likelihood of early death, so they might be expected to influence mortality calculations but not necessarily HRQoL outcomes. Another issue is that ICH location was assessed on varying thicknesses of axial slices of brain CT scans, which adds some noise to the data but probably not of sufficient magnitude to bias the observed changes in one particular site over another. There was a small number of infratentorial and lobar ICH, not allowing for further anatomical segmentation of these compartments. Despite the large size of the INTERACT dataset, when individual complete hemorrhage is analyzed the numbers are reduced to the point where less powerful effects no longer reach significance. Again, this affects mortality analysis more than disability for the reasons stated above.

We have shown that specific ICH sites, namely the posterior limb of the internal capsule, thalamus, and infratentorium, are associated with poor HRQoL in survivors. Infratentorial and thalamic involvement by ICH are associated with the greatest risk of death, and internal capsule lesions with the greatest residual disability. It is well-recognized that infratentorial location is a poor prognostic factor. To this, we can now add deep, posterior supratentorial locations involving the thalamus or posterior limb of internal capsule. These data offer additional practical help for clinicians in assessing patients with ICH and counseling patients and families, as well as in planning rehabilitation and developing individualized pathways of care.

## Supplementary Material

Data Supplement

Coinvestigators

## GLOSSARY

BP: blood pressure
CI: confidence interval
EQ-5D: European Quality of Life Scale
HRQoL: health-related quality of life
ICH: intracerebral hemorrhage
IQR: interquartile range
IVH: intraventricular hemorrhage
INTERACT2: Intensive Blood Pressure Reduction in Acute Cerebral Hemorrhage Trial
mRS: modified Rankin Scale
NIHSS: NIH Stroke Scale
OR: odds ratio
