# Supplementary material for: Intracerebral hemorrhage location and outcome among INTERACT2 participants
Source: Neurology. 2017 Apr 11;88(15):1408–14. doi: 10.1212/WNL.0000000000003771 (PMC5386433; doi:10.1212/WNL.0000000000003771)
Supplement: Data Supplement [file supp_WNL.0000000000003771_e-Tables.pdf]

Table e-1. Most common patterns of ICH (when present in more than 10 patients)

Number of patients	Single or multiple sites	Locations
342	Single	Putamen/globus pallidus
339	Multiple (2)	Thalamus and posterior limb of internal capsule
236	Multiple (3)	Posterior limb of internal capsule, putamen/globus pallidus and external capsule
181	Single	Thalamus
181	Single	Lobar
177	Multiple (2)	Putamen/globus pallidus and posterior limb of internal capsule
153	Multiple (2)	Putamen/globus pallidus and external capsule
138	Single	Infratentorial
50	Multiple (4)	Posterior limb of internal capsule, putamen/globus pallidus, external capsule, lobar
48	Multiple (3)	Thalamus, posterior limb of internal capsule, putamen/globus pallidus
28	Multiple (4)	Thalamus, posterior limb of internal capsule, putamen/globus pallidus and external capsule
19	Multiple (3)	Putamen/globus pallidus, external capsule and lobar
18	Multiple (3)	Anterior limb of internal capsule, putamen/globus pallidus, external capsule
18	Multiple (2)	Anterior limb of internal capsule and putamen/globus pallidus

Abbreviations: ICH = intracerebral hemorrhage.

869 ICH encompassed single locations and 1197 multiple locations.

Table e-2. Multivariable logistic regression analyses for associations between ICH sites and outcome

	Death or major disability		Major disability		Death	
	OR	95% CI	OR	95% CI	OR	95% CI
Age (yr)	1.05	1.04-1.06 [c]	1.01	1.01-1.02 [c]	1.05	1.04-1.06 [c]
Female	1.01	0.81-1.26	1.16	0.95-1.41	0.69	0.50-0.94 [a]
China region	0.61	0.47-0.79 [c]	0.69	0.55-0.86 [b]	0.96	0.68-1.36
Systolic BP (mm Hg)	1.00	1.00-1.01	1.00	0.99-1.00	1.02	1.01-1.03 [c]
High NIHSS (≥11 vs. <11)	4.20	3.30-5.34 [c]	2.53	2.02-3.17 [c]	3.21	2.14-4.81 [c]
Ischemic stroke	1.50	1.03-2.19 [a]	1.33	0.96-1.85	1.07	0.66-1.72
Diabetes	1.32	0.93-1.89	1.27	0.93-1.73	0.94	0.59-1.50
Antihypertensives	0.96	0.76-1.20	0.92	0.75-1.12	1.15	0.83-1.58
Lipid lowering agents	0.94	0.57-1.55	0.85	0.56-1.28	1.09	0.63-1.90
Antithrombotics	1.11	0.73-1.68	0.89	0.63-1.27	1.13	0.71-1.81
Intensive BP lowering treatment	0.95	0.77-1.18	0.94	0.78-1.14	1.00	0.74-1.34
Time from onset to CT (hr) [d]	0.81	0.68-0.96 [a]	0.83	0.71-0.97 [a]	1.03	0.80-1.32
Baseline ICH volume (mL) [d]	1.81	1.55-2.11 [c]	1.30	1.14-1.49 [c]	1.93	1.52-2.46 [c]

Intraventricular extension of ICH	1.15	0.85-1.56	0.87	0.67-1.13	1.25	0.84-1.85
Left hemisphere ICH	1.03	0.83-1.28	1.25	1.03-1.52 [a]	0.66	0.48-0.89 [b]
ICH location						
Caudate head	0.42	0.16-1.14	0.24	0.09-0.62 [b]	2.19	0.77-6.26
Thalamus	2.24	1.40-3.57 [c]	1.18	0.82-1.71	1.97	1.18-3.29 [b]
Putamen/globus pallidus	1.36	0.87-2.14	0.86	0.60-1.22	1.11	0.68-1.82
External capsule	1.05	0.78-1.40	0.96	0.74-1.25	1.23	0.81-1.87
Anterior limb of internal capsule	1.03	0.56-1.91	1.00	0.59-1.71	0.94	0.45-1.97
Posterior limb of internal capsule	2.10	1.65-2.68 [c]	1.81	1.45-2.26 [c]	1.04	0.72-1.51
Lobar	1.34	0.86-2.08	0.61	0.43-0.88 [b]	1.95	1.21-3.15 [b]
Infratentorial	3.04	1.68-5.50 [c]	1.27	0.77-2.11	2.45	1.09-5.50 [a]

[a] $0.01 \le p \le 0.05$, [b] $0.001 \le p < 0.01$, [c] $p < 0.001$, [d] Log-transformed

Abbreviations: ICH = intracerebral hemorrhage; OR= odds ratio, CI=confidence interval; BP = blood pressure; NIHSS = National Institutes of Health Stroke Scale; CT = computed tomography.

The c statistics for the models of death or major disability, major disability, and death were 0.83, 0.72, and 0.82, respectively.

Table e-3. Multivariable logistic regression analyses for associations between ICH location and HRQoL measured by the EQ-5D scale, by utility score (≤0.7 vs. >0.7) and dimension (some/moderate or severe problems vs. no problem)

	Utility score		Mobility		Self-care		Usual activity		Pain/discomfort		Anxiety/depression	
	**OR**	**95% CI**	**OR**	**95% CI**	**OR**	**95% CI**	**OR**	**95% CI**	**OR**	**95% CI**	**OR**	**95% CI**
Age (yr)	1.03	1.02-1.04 [c]	1.03	1.02-1.04 [c]	1.03	1.02-1.05 [c]	1.03	1.02-1.04 [c]	1.01	1.00-1.02	1.02	1.01-1.02 [b]
Female	1.23	0.98-1.55	1.04	0.82-1.31	1.08	0.85-1.37	1.04	0.81-1.33	1.29	1.04-1.58 [a]	1.08	0.87-1.34
China region	0.73	0.56-0.95 [a]	0.89	0.67-1.18	0.68	0.51-0.90 [b]	0.47	0.35-0.64 [c]	1.21	0.95-1.55	0.76	0.59-0.98 [a]
Systolic BP (mm Hg)	1.00	1.00-1.01	1.00	1.00-1.01	1.00	1.00-1.01	1.00	0.99-1.01	1.00	0.99-1.01	1.01	1.00-1.01
High NIHSS (≥11 vs. <11)	2.80	2.18-3.58 [c]	3.03	2.33-3.95 [c]	3.84	2.97-4.96 [c]	3.95	3.00-5.20 [c]	2.15	1.70-2.72 [c]	1.81	1.42-2.31 [c]
Ischemic stroke	1.09	0.73-1.62	1.38	0.91-2.08	1.40	0.93-2.11	1.01	0.66-1.53	0.82	0.58-1.18	0.96	0.66-1.39
Diabetes	1.07	0.74-1.55	1.17	0.80-1.72	1.35	0.92-1.98	1.28	0.85-1.93	0.74	0.53-1.03	0.89	0.63-1.26
Antihypertensives	1.07	0.85-1.35	0.88	0.70-1.11	0.98	0.77-1.24	0.95	0.74-1.21	1.13	0.91-1.39	1.20	0.96-1.49
Lipid lowering agents	0.95	0.56-1.62	0.87	0.51-1.50	0.68	0.39-1.18	0.71	0.39-1.29	1.48	0.94-2.34	1.09	0.68-1.73
Antithrombotics	1.93	1.25-2.98 [b]	1.30	0.82-2.06	1.26	0.80-1.9	2.03	1.22-3.38 [b]	1.09	0.74-1.60	1.40	0.94-2.06
Intensive BP lowering treatment	0.88	0.71-1.09	1.04	0.83-1.29	0.90	0.72-1.13	0.80	0.63-1.01	0.88	0.72-1.07	1.00	0.81-1.23
Time from onset to CT (hr) [d]	0.76	0.64-0.91 [b]	0.73	0.61-0.88 [c]	0.79	0.66-0.95 [a]	0.77	0.64-0.93 [b]	0.93	0.79-1.09	0.91	0.77-1.07
Baseline ICH volume (mL) [d]	1.81	1.53-2.14 [c]	1.54	1.32-1.79 [c]	1.84	1.55-2.18 [c]	1.63	1.39-1.92 [c]	1.20	1.04-1.38 [a]	1.41	1.21-1.64 [c]
Intraventricular extension of ICH	1.15	0.84-1.56	1.24	0.89-1.71	1.09	0.79-1.50	1.22	0.87-1.71	0.86	0.65-1.14	0.80	0.59-1.07
Left hemisphere ICH	1.01	0.80-1.26	1.06	0.84-1.33	1.01	0.80-1.27	1.26	0.99-1.59	0.89	0.73-1.09	0.97	0.78-1.20

ICH location												
Caudate head	0.26	0.08-0.82 [a]	1.59	0.57-4.47	0.28	0.09-0.89 [a]	0.32	0.10-1.00	0.60	0.23-1.60	0.24	0.07-0.83 [a]
Thalamus	2.14	1.32-3.48 [b]	2.42	1.45-4.05 [c]	2.28	1.37-3.82 [b]	3.00	1.69-5.31 [c]	0.90	0.60-1.35	1.31	0.86-1.99
Putamen/globus pallidus	1.27	0.80-2.02	1.34	0.81-2.20	1.24	0.76-2.02	1.59	0.92-2.76	0.82	0.55-1.21	0.98	0.66-1.48
External capsule	0.98	0.73-1.33	1.16	0.86-1.57	1.19	0.87-1.62	1.17	0.85-1.61	0.86	0.65-1.13	0.75	0.56-1.00
Anterior limb of internal capsule	1.33	0.70-2.53	0.72	0.38-1.35	1.12	0.58-2.18	0.79	0.40-1.55	1.11	0.63-1.95	1.50	0.83-2.72
Posterior limb of internal capsule	1.87	1.45-2.40 [c]	1.54	1.19-1.99 [b]	2.04	1.57-2.65 [c]	1.70	1.30-2.23 [c]	1.51	1.19-1.90 [c]	1.41	1.11-1.80 [b]
Lobar	1.18	0.74-1.87	1.23	0.74-2.03	1.08	0.66-1.76	1.51	0.88-2.62	0.71	0.47-1.08	1.03	0.67-1.57
Infratentorial	2.81	1.52-5.20 [b]	4.03	2.16-7.50 [c]	2.57	1.35-4.87 [b]	3.66	1.88-7.15 [c]	1.11	0.66-1.89	1.35	0.77-2.38
Proxy responder at 90-day HRQoL assessment	1.72	1.37-2.15 [c]	1.84	1.45-2.33 [c]	2.44	1.93-3.08 [c]	2.44	1.90-3.13 [c]	1.14	0.93-1.41	1.32	1.07-1.64 [a]

[a] 0.01≤p≤0.05, [b] 0.001≤p<0.01, [c] p<0.001, [d] Log-transformed

Abbreviations: ICH = intracerebral hemorrhage; HRQoL = Health-related Quality of Life; EQ-5D = European Quality of life five dimensions; OR= odds ratio, CI = confidence interval; BP = blood pressure; NIHSS = National Institutes of Health Stroke Scale; CT = computed tomography.

The c statistics for the models of utility score, mobility, self-care, usual activity, pain/discomfort and anxiety/depression were 0.79, 0.78, 0.83, 0.82, 0.66, and 0.69, respectively.

Table e-4. Multivariable logistic regression analyses for associations between ICH location and outcome (not adjusted for baseline NIHSS)

	**Death or major disability**		**Major disability**		**Death**	
	**OR**	**95% CI**	**OR**	**95% CI**	**OR**	**95% CI**
Caudate head	0.37	0.14-0.94 [b]	0.21	0.08-0.55 [b]	1.81	0.65-5.04
Thalamus	2.82	1.78-4.47 [c]	1.33	0.93-1.91	2.15	1.30-3.56 [b]
Putamen/globus pallidus	1.61	1.04-2.50 [b]	0.92	0.65-1.30	1.17	0.72-1.91
External capsule	1.10	0.83-1.45	0.99	0.77-1.28	1.24	0.82-1.88
Anterior limb of internal capsule	1.03	0.58-1.83	1.02	0.60-1.72	0.98	0.47-2.06
Posterior limb of internal capsule	2.18	1.73-2.75 [c]	1.90	1.53-2.36 [c]	1.14	0.79-1.64
Lobar	1.16	0.76-1.77	0.55	0.38-0.79 [b]	1.65	1.02-2.65 [a]
Infratentorial	3.67	2.03-6.61 [c]	1.40	0.85-2.31	2.27	1.02-5.06 [a]

[a] $0.01 \le p \le 0.05$, [b] $0.001 \le p < 0.01$, [c] $p < 0.001$.

Abbreviations: ICH = intracerebral hemorrhage; OR = odds ratio, CI =confidence interval.

Multivariable models were adjusted for age, female sex, China region, history of ischemic stroke and diabetes, medication history of antihypertensives, antithrombotics and lipid lowering agents, systolic blood pressure, onset to computed tomography time (log-transformed), baseline hematoma volume (log-transformed), caudate head, thalamus, putamen/globus pallidus, external capsule, anterior and posterior limbs of internal capsule, lobar, infratentorial, intraventricular extension of hematoma, ICH laterality and randomization to intensive blood pressure lowering.

Table e-5. Multivariable logistic regression analyses for associations between ICH location and HRQoL measured by the EQ-5D, by utility score (≤0.7 vs. >0.7) and dimension (some/moderate or severe problems vs. no problem; not adjusted for baseline NIHSS)

	Utility score		Mobility		Self-care		Usual activity		Pain/discomfort		Anxiety/depression	
	OR	95% CI	OR	95% CI	OR	95% CI	OR	95% CI	OR	95% CI	OR	95% CI
Caudate head	0.22	0.07-0.69 [b]	1.47	0.52-4.16	0.23	0.07-0.71 [a]	0.28	0.09-0.85 [a]	0.53	0.20-1.42	0.22	0.06-0.74 [a]
Thalamus	2.58	1.60-4.17 [c]	2.95	1.77-4.92 [c]	2.97	1.79-4.91 [c]	3.91	2.21-6.89 [c]	1.02	0.68-1.52	1.43	0.94-2.18
Putamen/globus pallidus	1.43	0.91-2.26	1.54	0.94-2.52	1.49	0.92-2.40	1.92	1.11-3.31 [a]	0.88	0.60-1.29	1.04	0.69-1.55
External capsule	1.04	0.77-1.39	1.22	0.91-1.64	1.25	0.93-1.68	1.25	0.92-1.69	0.90	0.69-1.17	0.77	0.58-1.03
Anterior limb of internal capsule	1.32	0.71-2.45	0.73	0.40-1.34	1.12	0.60-2.11	0.81	0.43-1.54	1.11	0.64-1.95	1.51	0.84-2.71
Posterior limb of internal capsule	1.92	1.50-2.46 [c]	1.61	1.25-2.07 [c]	2.09	1.62-2.68 [c]	1.76	1.36-2.28 [c]	1.56	1.24-1.96 [c]	1.45	1.14-1.85 [b]
Lobar	1.03	0.65-1.63	1.13	0.69-1.86	0.95	0.59-1.52	1.38	0.81-2.35	0.66	0.44-0.99 [a]	0.95	0.62-1.45
Infratentorial	3.18	1.71-5.89 [c]	5.05	2.70-9.45 [c]	3.06	1.61-5.81 [c]	4.80	2.46-9.38 [c]	1.22	0.72-2.07	1.43	0.81-2.52

[a] 0.01≤p≤0.05, [b] 0.001≤p<0.01, [c] p<0.001.

Abbreviations: ICH = intracerebral hemorrhage; HRQoL = Health-related Quality of Life; EQ-5D = European Quality of life five dimensions; NIHSS = National Institutes of Health Stroke Scale; OR= odds ratio; CI = confidence interval.

Multivariable models were adjusted for age, female sex, China region, history of ischemic stroke and diabetes, medication history of antihypertensives, antithrombotics and lipid lowering agents, systolic blood pressure, onset to computed tomography time (log-transformed), baseline hematoma volume (log-transformed), caudate head, thalamus, putamen/globus pallidus, external capsule, anterior and posterior limbs of internal capsule, lobar, infratentorial, intraventricular extension of hematoma, ICH laterality, randomization to intensive blood pressure lowering management policy, and proxy responders.

Table e-6. Hematoma volume by site of involvement

ICH site	N	Median (IQR) [a]
Caudate	42	14.7 (7.4-26.4)
Thalamus	640	7.6 (4.3-12.1)
Putamen/globus pallidus	1161	13.8 (8.3-22.0)
External capsule	553	16.2 (10.9-27.3)
Anterior limb of internal capsule	102	18.0 (10.5-31.3)
Posterior limb of internal capsule	957	11.0 (6.2-19.1)
Lobar	297	25.4 (13.7-43.1)
Infratentorial	141	3.3 (1.5-7.0)

[a] ICH volume in mL.

Abbreviations: ICH = intracerebral hemorrhage; N = number of cases by ICH site; IQR = interquartile range.

Table e-7. Model fit statistics for table 2 and 3 (main tables and supplemental tables)

Primary analysis models	Sensitivity [a]	Specificity [a]	PPV [a]	NPV [a]	AUC
Death or major disability	76.3	71.4	75.3	72.4	0.83
Major disability	49.6	75.3	58.5	68.1	0.72
Death	17.2	98.6	62.3	89.5	0.82
EQ-5D utility score	64.9	74.8	67.8	72.3	0.79
Mobility	83.9	49.4	75.6	62.1	0.78
Self-care	72.4	74.6	72.9	74.1	0.83
Usual activity	82.3	61.6	78.7	66.9	0.82
Pain and discomfort	36.2	79.7	54.4	65.2	0.66
Anxiety and depression	26.0	88.5	54.3	69.5	0.69

[a] For predicted probability cut-off of 0.5.
Abbreviations: PPV = positive predictive value; NPV = negative predictive value; AUC = area under the receiver operating characteristic curve; EQ-5D = European Quality of life five dimensions.
