# Supplementary material for: Intracerebral hemorrhage location and outcome among INTERACT2 participants
Source: Neurology. 2017 Apr 11;88(15):1408–14. doi: 10.1212/WNL.0000000000003771 (PMC5386433; doi:10.1212/WNL.0000000000003771)
Supplement: Data Supplement [file supp_WNL.0000000000003771_Figure_e-1.pdf]

Figure e-1. Patient selection flow chart

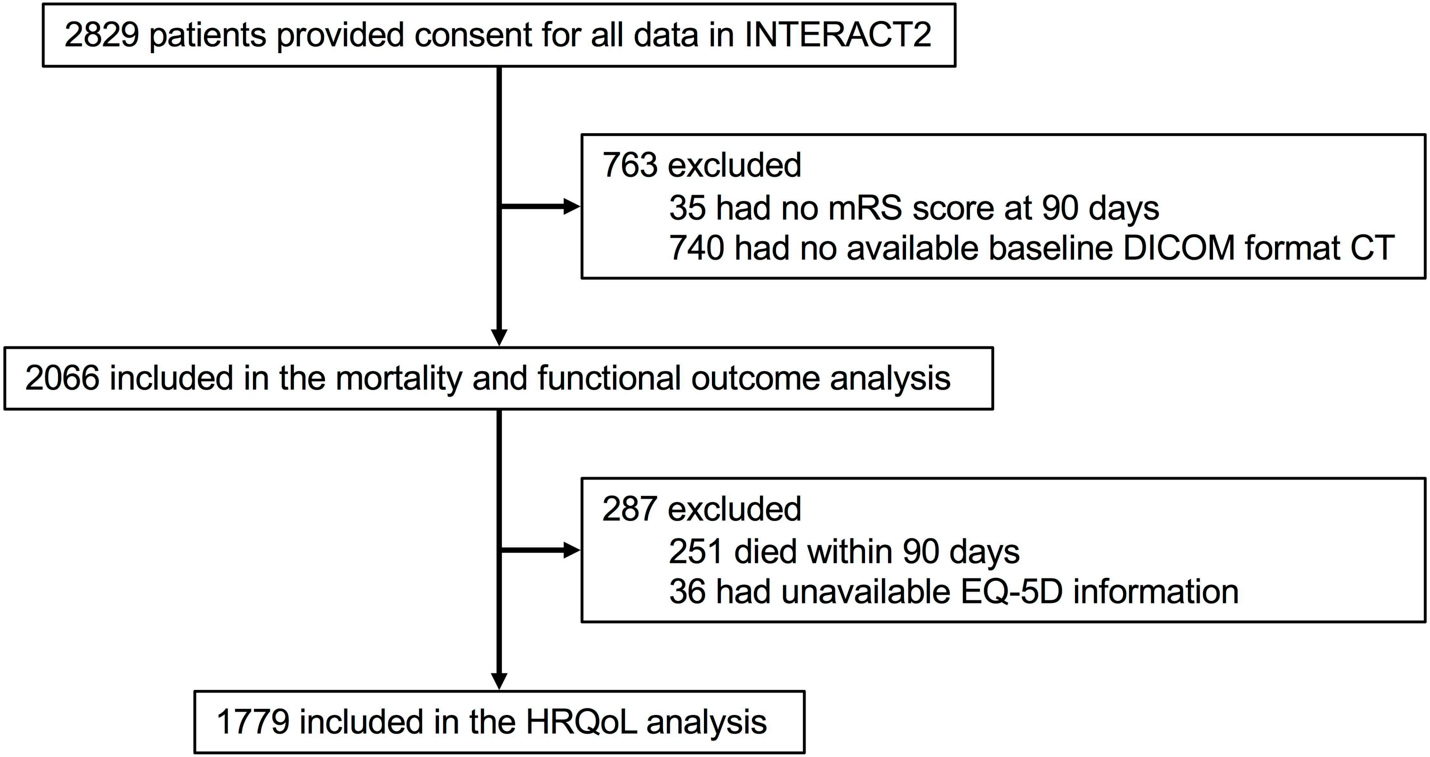

Abbreviations: INTERACT2 = the main phase of Intensive Blood Pressure Reduction in Acute Cerebral Hemorrhage Trial; mRS = modified Rankin Scale; DICOM = Digital Imaging and COmmunication in Medicine; CT = computed tomography; EQ-5D = European Quality of Life Scale; HRQoL = Health-related Quality of Life.
