# Supplementary material for: Intracerebral hemorrhage location and outcome among INTERACT2 participants
Source: Neurology. 2017 Apr 11;88(15):1408–14. doi: 10.1212/WNL.0000000000003771 (PMC5386433; doi:10.1212/WNL.0000000000003771)
Supplement: Coinvestigators [file supp_88_15_1408_v2_index.html]

Intracerebral hemorrhage location and outcome among INTERACT2 participants — Coinvestigators 

# Intracerebral hemorrhage location and outcome among INTERACT2 participants

## Coinvestigators

**Neurology® data supplements are not copyedited before publication. Published editorials and translations have been copyedited.  
 © 2017 American Academy of Neurology.  
  
 Files in this Data Supplement:**

- Coinvestigators - Microsoft Word file
