# Supplementary material for: Intracerebral hemorrhage location and outcome among INTERACT2 participants
Source: Neurology. 2017 Apr 11;88(15):1408–14. doi: 10.1212/WNL.0000000000003771 (PMC5386433; doi:10.1212/WNL.0000000000003771)
Supplement: Coinvestigators [file supp_WNL.0000000000003771_Coinvestigators.docx]

**INTERACT2 COINVESTIGATORS**

**Executive Committee:** C.S. Anderson (principal investigator), J. Chalmers (chair), H. Arima, S. Davis, E. Heeley, Y. Huang, P. Lavados*,* B. Neal, M.W. Parsons, R. Lindley, L. Morgenstern, T. Robinson, C. Stapf, C. Tzourio, J.G. Wang. **National Leaders:** *China* - *Steering Committee:* Y. Huang (chair), S. Chen, X.Y. Chen, L. Cui, Z. Liu, C. Lu, J. Wang, S. Wu, E. Xu, Q. Yang, C. Zhang, J. Zhang. *Europe* - *Austria*: R. Beer, E. Schmutzhard; *Belgium:* P. Redondo; *Finland*: M. Kaste, L. Soinne, T. Tatlisumak; *France*: C. Stapf; *Germany*: K. Wartenberg; *Italy*: S. Ricci; *The* *Netherlands:* K. Klijn; *Portugal*: E. Azevedo; *Spain*: A. Chamorro; *Switzerland*: M. Arnold, U. Fischer; *India:* S. Kaul, J. Pandian, H. Boyini, S. Singh; *North America –* A.A. Rabinstein; *South America - Argentina –* C. Estol; *Brazil –* G. Silva; *Chile –* P. Lavados, V.V. Olavarria; and *United Kingdom –* T.G. Robinson. **Data Safety and Monitoring Committee:** R.J. Simes (chair), M.-G. Bousser, G. Hankey, K. Jamrozik (deceased), S.C. Johnston, and S. Li. **Project Office Operations Committee:** E. Heeley (study director), C.S. Anderson, K. Bailey, J. Chalmers, T. Cheung, C. Delcourt, S. Chintapatla, E. Ducasse, T. Erho, J. Hata, B. Holder, E. Knight, R. Lindley, M. Leroux, T. Sassé, E. Odgers, R. Walsh, and Z. Wolfowicz. **Endpoint Adjudication Committee:** C.S. Anderson, G. Chen, C. Delcourt, S. Fuentes, R. Lindley, B. Peng, H.-M. Schneble, and M.-X. Wang. **Statistical Analysis:** H. Arima, L. Billot, S. Heritier, Q. Li, and M. Woodward. **CT Analyses:** C. Delcourt (chair), S. Abimbola, S. Anderson, E. Chan, G. Cheng, P. Chmielnik, J. Hata, S. Leighton, J.-Y. Liu, B. Rasmussen, A. Saxena, and S. Tripathy. **Data Management and Programming:** M. Armenis, M.A. Baig, B. Naidu, G. Starzec, and S. Steley. **Coordinating Centers:** *International* (The George Institute for Global Health, Sydney, Australia) – C.S. Anderson, E. Heeley, M. Leroux, C. Delcourt, T. Sassé, E. Knight, K. Bailey, T. Cheung, E. Odgers, E. Ducasse, B. Holder, Z. Wolfowicz, R. Walsh, S. Chintapatla, T. Erho; *Argentina*, *Buenos* *Aires* (STAT Research) – C. Estol, A. Moles, A. Ruiz, M. Zimmermann; *Brazil, Fortaleza* (Medicamenta MRS) – J. Marinho, S. Alves, R. Angelim, J. Araujo, L. Kawakami; *Chile,* *Santiago* (Clínica Alemana, Universidad del Desarrollo) – P. Lavados, V.V. Olavarria, C. Bustos, F. Gonzalez, P. Munoz Venturelli; *China, Beijing* (The George Institute China incorporating George Clinical, and Peking University First Hospital) – Y. Huang, X. Chen, Y. Huang, R. Jia, N. Li, S. Qu, Y. Shu, A. Song, J. Sun, J. Xiao, and Y. Zhao; *China, Shanghai* (The Centre for Epidemiological Studies and Clinical Trials, The Shanghai Institute of Hypertension, Rui Jin Hospital, Shanghai Jiaotong University School of Medicine) - J.G. Wang, Q. Huang; *Europe, Paris* (Unité de Recherche Clinique, APHP - Hôpital Lariboisière) – C. Stapf, E. Vicaut, A. Chamam, M.-C. Viaud, C. Dert, U. Fiedler, V. Jovis, S. Kabla, S. Marchand, A. Pena, V. Rochaud; *India, Hyderabad* (The George Institute India) – K. Mallikarjuna H. Boyini, N. Hasan; *Norway, Oslo* (Oslo University Hospital) – E. Berge, E.C. Sandset, A.S. Forårsveen; *United Kingdom* (Department of Cardiovascular Sciences, University of Leicester) – T. Robinson, D. Richardson, T. Kumar, S. Lewin; *United Kingdom* (London, Imperial Clinical Trials Unit) - N. Poulter, J. Field, A. Anjum, A. Wilson. **Principal Investigators and Coordinators (according to country and center, with** **numbers of patients in parentheses)**: *Argentina -* Clínica Instituto Medico Adrogue (1): H. Perelmuter, A.M. Agarie; Hospital Central de Mendoza (3): A.G. Barboza, L.A. Recchia, I.F. Miranda, S.G. Rauek, R.J. Duplessis; *Australia -* Austin Hospital (5): H. Dewey, L. Walker, S. Petrolo; Box Hill (3): C. Bladin; Gosford Hospital (22): J. Sturm, D. Crimmins, D. Griffiths, A. Schutz, V. Zenteno; John Hunter Hospital (10): M.W. Parsons, F. Miteff, N. Spratt, E. Kerr, C.R. Levi; Monash Medical Centre (5): T.G. Phan, H. Ma, L. Sanders, C. Moran, K. Wong; Royal Brisbane and Women's Hospital (3): S. Read, R. Henderson, A. Wong, R. Hull, G. Skinner; Royal Melbourne Hospital (13): S. Davis, P. Hand, B. Yan, H. Tu, B. Campbell; Royal Prince Alfred Hospital (4): C.S. Anderson, C. Delcourt; Sir Charles Gairdner Hospital (4): D.J. Blacker; Western Hospital (4): T. Wijeratne, M. Pathirage, M. Jasinararchchi, Z. Matkovic, S. Celestino; *Austria -* Allgenmeines Krankenhaus Linz (9): F. Gruber, M.R. Vosko, E. Diabl, S. Rathmaier; Innsbruck Medical University - Department of Neurology (34): R. Beer, E. Schmutzhard, B. Pfausler, R. Helbok; Medical University of Graz, Department of Neurology (20): F. Fazekas, R. Fischer, B. Poltrum, B. Zechner, U. Trummer; *Belgium -* Cliniques De L'Europe (Europe Clinic) (1): M.P. Rutgers; UCL St Luc (5): A. Peeters, A. Dusart, M.-C. Duray, C. Parmentier, S. Ferrao-Santos; Universitair Ziekenhuis Brussel (6): R. Brouns, S. De Raedt, A. De Smedt, R.-J. VanHooff, J. De Keyser; *Brazil (17) -* Hospital das Clínicas de Porto Alegre (5): S.C.O. Martins, A.G. de Almeida, R. Broudani, N.F. Titton; Hospital Quinta D'Or (1): G.R. de Freitas, F.M. Cardoso, L.M. Giesel, N.A. Lima Junior; Hospital Santa Marcelina (3): A.C. Ferraz de Almeida, R.B. Gomes, T.S. Borges dos Santos, E.M. Veloso Soares, O.L.A. Neto; Universidade Federal de São Paulo (7): G.S. Silva, D.L. Gomes, F.A. de Carvalho, M. Miranda, A. Marques; Universidade Federal do Paraná (1): V.F. Zétola, G. de Matia, M.C. Lange; *Chile -* Clinica Alemana de Santiago (8): J. Montes, A. Reccius, P. Munoz Venturelli, V.V. Olavarria, A. Soto; Clínica Alemana de Temuco, Chile (3): R. Rivas, C. Klapp; Clínica Dávila (5): S. Illanes, C. Aguilera, A. Castro; Complejo Asistencial Dr. Víctor Ríos Ruiz (12): C. Figueroa, J. Benavides, P. Salamanca, M.C. Concha, J. Pajarito; Hospital Naval Almirante Nef (1): P. Araya, F. Guerra; *China -* Baotou Central Hospital (225): Y. Li, G. Liu, B. Wang, J. Zhang, Y. Chong; Beijing Shijitan Hospital (19): M. He, L. Wang, J. Liu; Beijing Tongren Hospital (11): X. Zhang, C. Lai, H. Jiang, Q. Yang, S. Cui; Chang Ning District Central Hospital (25): Q. Tao, Y. Zhang, S. Yao, M. Xu, Y. Zhang; Changsha Central Hospital (42): Z. Liu, H. Xiao, J. Hu, J. Tang; Gongli Hospital, Pudong New Area, Shanghai (11): J. Sun, H. Ji, M. Jiang; Haidian Hospital, Beijing (9): F. Yu, Y. Zhang, X. Yang, X. Guo; Hejian City People's Hospital (158): Y. Wang, L. Wu, Z. Liu, Y. Gao, D. Sun; Hunan Province Brain Hospital (14): X. Huang, Y. Wang, L. Liu, Y. Li, P. Li; Jiangsu Province Hospital of Traditional Chinese Medicine (9): Y. Jiang, H. Li, H. Lu; Nanjing First Hospital (10): J. Zhou, C. Yuan; Navy General Hospital (2): X. Qi, F. Qiu, H. Qian, W. Wang, J. Liu; Peking University First Hospital (4): Y. Huang, W. Sun, F. Li, R. Liu, Q. Peng; Peking University Shougang Hospital (8): Z. Ren, C. Fan, Y. Zhang, H. Wang, T. Wang; People's Hospital of Beijing Daxing District (60): F. Shi, C. Duan, S. Chen, J. Wang, Z. Chen; Pinggu County Hospital, Beijing (60): X. Tan, Z. Zhao, Y. Gao, J. Chen, T. Han; Qinghai Province People's Hospital (12): S. Wu, L. Zhang, L. Wang, Q. Hu, Q. Hou; Qinghai University Affiliated Hospital (41): X. Zhao, L. Wang, G. Zeng, L. Ma, F. Wang; Ruijin Hospital Affiliated to Shanghai Jiaotong University School of Medicine (4): S. Chen, L. Zeng, Z. Guo, Y. Fu, Y. Song; Second Hospital of Hebei Medical University (83): L. Tai, X. Liu, X. Su, Y. Yang, R. Dong; Shijiazhuang 260 Hospital (25): Y. Xu, S. Tian, S. Cheng, L. Su, X. Xie; The Affiliated Hospital of Xuzhou Medical College (39): T. Xu, D. Geng, X. Yan, H. Fan, N. Zhao; The Branch Hospital of the First People's Hospital (52): S. Wang, J. Yang; The Chinese PLA No. 263 Hospital (108): J. Zhang, M. Yan, L. Li; The Fifth Affiliated Hospital Sun Yat-Sen University (26): Z. Li, X. Xu, F. Wang; The First Affiliated Hospital of Baotou Medical College (81): L. Wu, X. Guo, Y. Lian, H. Sun, D. Liu; The First Affiliated Hospital of Fujian Medical University (12): N. Wang, Q. Tang; The First Affiliated Hospital of Wenzhou Medical College (68): Z. Han, L. Feng; The Fourth Hospital of Jilin University (73): Y. Cui, J. Tian, H. Chang, X. Sun, J. Wang; The Second Affiliated Hospital Suzhou University (31): C. Liu, Z. Wen; The Second Affiliated Hospital of Guangzhou Medical College (38): E. Xu, Q. Lin; The Second Affiliated Hospital of Wenzhou Medical College (21): X. Zhang, L. Sun, B. Hu, M. Zou, Q. Bao; The Second Hospital of Qinghuangdao (51): X. Lin, L. Zhao, X. Tian, H. Wang, X. Wang; The Second Hospital of Tianjin Medical University (12): X. Li, L. Hao, Y. Duan, R. Wang, Z. Wei; Third Hospital of Hebei Medical University (20): J. Liu, S. Ren, H. Ren, Y. Wang, Y. Dong; Tianjin Medical University General Hospital (27): Y. Cheng, M. Zou, W. Liu, J. Han, C. Zhang; Tianjin Third Central Hospital (14): Z. Zhang, J. Zhu, Y. Wang, Q. Li; Traditional Chinese Medicine Hospital, Zhangjiagang (10): J. Qian, Y. Sun, K. Liu, F. Long; Wangcheng County People's Hospital of Hunan Province (8): X. Peng, Q. Zhang, Z. Yuan, C. Wang, M. Huang; Wuxi People's Hospital (5): J. Zhang, F. Wang, P. He, Y. You, X. Wang; Xiangya Hospital Central-South University (8): Q. Yang, H. Wang, J. Xia, L. Zhou, Y. Hou; Xining First People's Hospital (21): Y. Wang, L. Liu, Y. Qi, L. Mei, R. Lu; Xuzhou Central Hospital (128): G. Chen, L. Liu, L. Ping, W. Liu, S. Zhou; Yutian County Hospital, Hebei Province (225): J. Wang, L. Wang, H. Li, S. Zhang, L. Wang; Zengcheng People's Hospital (15): R. Zou, J. Guo, M. Li, W. Wei; *Finland* - Helsinki University Central Hospital (36): L. Soinne, S. Curtze, M. Saarela, D. Strbian, F. Scheperjans; *France -* Centre Hospitalier de Saint Denis – Hôpital Delafontaine (11): T. De Broucker, C. Henry, R. Cumurciuc, N. Ibos-Augé; Centre Hospitalier de Versailles André-Mignot (16): A.-C. Zéghoudi, F. Pico; CH Calais (12): O. Dereeper, M.-C. Simian, C. Boisselier, A. Mahfoud; CHRU de Brest (6): S. Timsit, F.M. Merrien; CHU de Nantes - Hôpital G&R Laënnec (13): B. Guillon, M. Sevin, F. Herisson, C. Magne; Hôpital de Meaux (12): A. Ameri, C. Cret, S. Stefanizzi, F. Klapzcynski; Hôpital Kremlin Bicêtre (7): C. Denier, M. Sarov-Riviere; Hôpital Lariboisière (37): C. Stapf, P. Reiner, J. Mawet, D. Hervé, F. Buffon; Hôpital Ste-Anne (9): E. Touzé, V. Domigo, C. Lamy, D. Calvet, M. Pasquini; Hôpital Tenon (12): S. Alamowitch, P. Favrole, I.-P. Muresan; Pitié Salpêtrière (37): S. Crozier, C. Rosso, C. Pires, A. Leger, S. Deltour; Roger Salengro Lille (30): C Cordonnier, H. Henon, C. Rossi; Service de Neurologie et Neurovasculaire, Groupe Hospitalier Paris Saint Joseph (19): M. Zuber, M. Bruandet, R. Tamazyan, C. Join-Lambert; *Germany -* Charité-University Medicine Berlin - Center for Stroke Research Berlin (CSB) (46): E. Juettler, T. Krause, S. Maul, M. Endres, G.J. Jungehulsing; Department of Neurology University of Heidelberg UMM Mannheim (23): M. Hennerici, M. Griebe, T. Sauer, K. Knoll; Department of Neurology, University of Ulm (8): R. Huber, K. Knauer, C. Knauer, S. Raubold; Dresden University of Technology, University Hospital, Department of Neurology (27): H. Schneider, H. Hentschel, C. Lautenschläger, E. Schimmel, I. Dzialowski; Goethe University Hospital Frankfurt (10): C. Foerch, M. Lorenz, O. Singer, I.M. R. Meyer dos Santos; Klinikum Frankfurt (Oder) (1): A. Hartmann, A. Hamann, A. Schacht, B. Schrader, A. Teíchmann; Martin Luther University (21): K.E. Wartenberg, T.J. Mueller; University Hospital Düsseldorf (3): S. Jander, M. Gliem, C. Boettcher; University Medical Center Hamburg – Eppendorf (2): M. Rosenkranz, C. Beck, D. Otto, G. Thomalla, B. Cheng; *Hong Kong -* Prince of Wales Hospital, Chinese University of Hong Kong (1): K.S. Wong, T.W. Leung, Y.O.Y. Soo; *India -* Apollo Hospitals (1): S. Prabhakar, S.R. Kesavarapu, P.K. Gajjela, R.R. Chenna; Baby Memorial Hospital (13): K. Ummer, M. Basheer, A. Andipet; CARE Hospital, Nampally (10): M.K.M. Jagarlapudi, A.U.R. Mohammed, V.G. Pawar, S.S.K. Eranki; Christian Medical College & Hospital (17): J. Pandian, Y. Singh, N. Akhtar; GNRC Hospitals (16): N.C. Borah, M. Ghose, N. Choudhury; Jehangir Clinical Development Centre Pvt Ltd (2): N.R. Ichaporia, J. Shendge, S. Khese; Lalitha Super Specialities Hospital (27): V. Pamidimukkala, P. Inbamuthaiah, S.R. Nuthakki, N.M.R. Tagallamudi, A.K. Gutti; Postgraduate Institute of Medical Education & Research (10): D. Khurana, P. Kesavarapu, V. Jogi, A. Garg, D. Samanta; St. John's Medical College & Hospital (1): G.R.K. Sarma, R. Nadig, T. Mathew, M.A. Anandan; *Italy -* Central follow up for Italy: E. Caterbi; Nuovo Ospedale Civile, AUSL Modena (15): A. Zini, M. Cavazzuti, F. Casoni, R. Pentore, F. Falzone; Ospedale di Branca (6): S. Ricci, T. Mazzoli, L.M. Greco, C. Menichetti, F. Coppola; Ospedale di Città di Castello (16): S. Cenciarelli, E. Gallinella, A. Mattioni, R. Condurso, I. Sicilia; San Giovanni Battista (4): M. Zampolini, F. Corea, M. Barbi, C. Proietti; Sapienza University Unità di Trattamento Neurovascolare (6): D. Toni, A. Pieroni, A. Anzini, A. Falcou, M. Demichele; *The Netherlands -* University Medical Center Utrecht (2): C.J.M. Klijn; *Norway -* Sørlandet Sykehus HF Kristiansand (2): A. Tveiten, E.T. Thortveit, S. Pettersen; Sykehuset Innlandet HF Lillehammer (2): N. Holand, B. Hitland; University Hospital North Norway (1): S.H. Johnsen, A. Eltoft; *Pakistan –* Aga Khan University (9): M. Wasay, A. Kamal, A. Iqrar, L. Ali, D. Begum; *Portugal (22) -* Centro Hospitalar Sao Joao (21): G. Gama, E. Azevedo, L. Fonseca, G. Moreira; Centro Hospitalar Vila Nova de Gaia (1): L.M. Veloso, D. Pinheiro, L. Paredes, C. Rozeira, T. Gregorio; *Spain -* Complejo Hospitalario Universitario de Albacete (6): T. Segura Martin, O. Ayo, J. Garcia-Garcia, I. Feria Vilar, I. Gómez Fernández; Hospital Clinico de Barcelona (10): A. Chamorro, S. Amaro, X. Urra, V. Obach, A. Cervera; Hospital Universitari de Girona, Dr Josep Trueta (34): Y. Silva, J. Serena, M. Castellanos, M. Terceno, C. Van Eendenburg; *Switzerland -* University of Bern, Inselspital (3): U. Fischer, M. Arnold, A. Weck, O. Findling, R. Lüdi; *United Kingdom -* Addenbrookes Hospital (1): E.A. Warburton, D. Day, N. Butler, E. Bumanlag; Bristol Royal Infirmary (1): S. Caine, A. Steele, M. Osborn, E. Dodd, P. Murphy; County Durham & Darlington NHS Foundation Trust (1): B. Esisi, E. Brown, R. Hayman, V. K.V. Baliga, M. Minphone; John Radcliffe Hospital (3): J. Kennedy, I. Reckless, G. Pope, R. Teal, K. Michael; King's College Hospital (8): D. Manawadu, L. Kalra, R. Lewis, B. Mistry, E. Cattermole; Leeds General Infirmary (2): A. Hassan, L. Mandizvidza, J. Bamford, H. Brooks, C. Bedford; Musgrove Park Hospital (1): R. Whiting, P. Baines, M. Hussain, M. Harvey; New Cross Hospital (4): K. Fotherby, S. McBride, P. Bourke, D. Morgan, K. Jennings-Preece; Northumbria Healthcare – Wansbeck and North Tyneside General Hospitals (2): C. Price, S. Huntley, V.E. Riddell, G. Storey, R.L. Lakey; Nottingham University Hospital (2): G. Subramanian; Royal Bournemouth Hospital (3): D. Jenkinson, J. Kwan, O. David, D. Tiwari; Royal Devon and Exeter Hospital (3): M. James, S. Keenan, H. Eastwood; Royal United Hospital Bath NHS Trust (6): L. Shaw, P. Kaye, D. Button, B. Madigan, D. Williamson; Royal Victoria Infirmary Hospital NHS Foundation Trust (8): A. Dixit, J. Davis, M.O. Hossain, G.A. Ford; Salford Royal NHS Foundation Trust (12): A. Parry-Jones, V. O'Loughlin, R. Jarapa, Z. Naing; St George's Healthcare NHS Trust (1): C. Lovelock, J. O'Reilly, U. Khan; St. Thomas Hospital (1): A. Bhalla, A. Rudd, J. Birns; University College London Hospitals NHS Foundation Trust (6): D.J. Werring, R. Law, R. Perry, I. Jones, R. Erande; University Hospital of North Staffordshire (2): C. Roffe, I. Natarajan, N. Ahmad, K. Finney, J. Lucas; University Hospitals of Leicester NHS Trust (3): A. Mistri, D. Eveson, R. Marsh, V. Haunton, T. Robinson; *USA* *-* Mayo Clinic (11): A.A. Rabinstein, J.E. Fugate, S.W. Lepore.
